# Reimagining hospital management: the balanced scorecard as a catalyst for employee retention and organizational excellence

**DOI:** 10.3389/fpubh.2024.1485683

**Published:** 2024-12-11

**Authors:** Feng Guo, Ying Sophie Huang, Moeki Nemoto

**Affiliations:** ^1^School of Innovation and Entrepreneurship, Zhejiang Shuren University, Hangzhou, China; ^2^Department of Finance and Accounting, School of Management & Capital Market Research Center, Zhejiang University, Hangzhou, China; ^3^Faculty of Economics, The International University of Kagoshima, Kagoshima, Japan; ^4^School of Management, Zhejiang University, Hangzhou, China

**Keywords:** balanced scorecard, hospital management, employee turnover, communication, engagement, healthcare workforce

## Abstract

Employee turnover in healthcare institutions is a critical issue affecting both quality of care and organizational costs. This study examines the potential impact of the Balanced Scorecard (BSC) as a communication tool on employee turnover rates in a Japanese hospital setting. A case study of Bethlehem Garden Hospital in Tokyo, Japan, was conducted to examine turnover rates before and after the implementation of BSC. The study also compares these rates to industry averages in Japan and the United States. The results show a significant reduction in turnover rates from 23.6% in 2015 to 3.4% in 2023 following the implementation of BSC, which is lower than both national and international industry averages. This reduction corresponded with increased employee engagement scores. The study suggests that the BSC when effectively implemented and communicated, can help reduce turnover by improving organizational alignment, employee engagement, and trust. Although the single case study design limits generalizability, these findings provide valuable insights into the potential of BSC as a strategic tool for addressing turnover in healthcare settings. Future research directions are suggested to validate these findings further in diverse healthcare environments.

## 1 Introduction

Decreasing turnover rates in healthcare institutions is an important issue worldwide (1). In particular, pandemics such as COVID-19 have increased the need to prevent turnover among healthcare workers (2). High hospital turnover leads to lower quality of care (3–5) and increased financial burden on hospitals (4–7). De Vries et al. (2) highlight that distinctive interventions consistent with mission, vision, and values are needed to improve the retention of healthcare workers. The Balanced Scorecard (BSC) is useful in addressing these issues. The BSC is a management and communication tool that communicates the mission, vision, values, and strategy (8, 9). Recently, studies have linked BSC as a corporate communication tool [e.g. (10–12)]. Corporate communication is one of the communication theories in organizations, and its purpose is to manage the relationship between the organization and its stakeholders (13).

Hospitals in Japan face increasing pressure to enhance their management practices. In other words, sustainable management is necessary. A June 2021 survey by the Japan Hospital Federation (14) revealed that about 76.9% of the hospitals surveyed suffered from financial losses. The impacts caused by hospital deficits are manifold. Hospitals under financial pressure have a negative impact on the quality of care and patient safety (15). Dubas-Jakóbczyk et al. (16) discuss how physician shortages in Polish hospitals can severely impact hospital management. Hospital financial losses can make it difficult to improve physician salaries and benefits and can reduce the attractiveness of hospitals, for example, by forcing them to stop providing certain services due to a shortage of physicians. Thus, hospital deficits are a factor that affects not only hospitals but also patient health and community healthcare. BSC has emerged as a promising tool for hospital management in Japan in this challenging environment. Healthcare institutions widely adopt BSC to improve the quality of care (17, 18).

This paper focuses on turnover as an indicator of quality of care. Turnover rates in healthcare are an important indicator associated with quality of care and patient safety (4, 19–22). Several studies link BSC to turnover rates [e.g., (12, 23–25)]. However, only a few studies, such as Huang et al. (12), present data on reduced turnover.

Huang et al. (12) conducted a case study of BSC as a communication tool at Bethlehem Garden Hospital in Kiyose, Tokyo, Japan. The results showed that the implementation of BSC led to a decrease in turnover rates. However, the data presented here is limited to Bethlehem Garden Hospital and does not compare turnover rates with those of other hospitals in Japan or in the same industry.

This paper will focus on this Japanese data to provide additional insight into whether BSC can reduce turnover. Specifically, we will add data on turnover rates in the healthcare and welfare industries from Japan’s Ministry of Health, Labour and Welfare (MHLW) and nursing staff turnover rates from the Japan Nursing Association (JNA). Data on hospital turnover rates in the U.S. will also be presented for reference. In addition, the changes in the operational status of Bethlehem Garden Hospital from the implementation of the BSC to 2023 will be described. This will allow us to evaluate the Bethlehem Garden Hospital case study properly results in terms of lower turnover rates. If BSC can be shown to be effective in reducing turnover, its usefulness in healthcare institutions will increase.

## 2 Research methods

This paper is similar to the case study by Huang et al. (12), where data triangulation (interviews, action research, and document analysis) was conducted. This study employs an abductive research approach to analyze how BSC functions as a communication tool in healthcare settings. Our research design utilizes a single case study method to enable in-depth exploration and theory development.

### 2.1 Semi-structured interviews

Phase 1: Initial face-to-face interview (May 18, 2018, 3:00–5:20 pm) with the Administrative Director to gather baseline information about BSC implementation and strategy. Phase 2: Follow-up email interviews from 2022 onwards to collect updated information (adapted due to COVID-19 restrictions).

### 2.2 Action research

Period: 2018–2024. Focus: Enhancement of BSC and its dissemination among hospital staff. Key activities: Three training sessions (June–July 2019), development of departmental BSC, integration of SDGs into BSC framework.

### 2.3 Document analysis

External materials to capture communication outside the organization (2016–2024): website content, blog posts, PR magazines, third-party evaluation reports, and papers by hospital personnel.

Our analysis followed an abductive approach, which involved iterating between existing theories and empirical findings while maintaining openness to new theoretical insights. “Abduction refers to a creative inferential process aimed at producing new hypotheses and theories based on surprising research evidence” (26).

We employed multiple validation strategies to ensure research quality and trustworthiness, including data triangulation, multiple researcher verification, and regular stakeholder discussions throughout our longitudinal observation period from 2016 to 2024.

## 3 Bethlehem Garden Hospital case study overview

Bethlehem Garden Hospital, located in Kiyose City, Tokyo, Japan, provides an intriguing case study for examining the potential impact of the BSC on employee turnover rates in healthcare institutions. This long-term care hospital, with a capacity of 96 beds, has a rich history dating back to its founding in October 1933 as a sanatorium farm. It obtained its hospital license in April 1935 and initially specialized in tuberculosis treatment. As of January 1, 2021, the hospital employed 199 staff members, comprising 90 full-time and 109 part-time employees.

The hospital operates under the ownership of the social welfare corporation Jiseikai, which manages various educational and social service institutions. Bethlehem Garden Hospital adheres to a Christian Catholic philosophy, encapsulated in its mission statement: “We inherit the will of Father Joseph Flaujac, our founder, and faithfully provide warm medical care for the sick according to the spirit of Christ’s love.” This mission is supported by seven fundamental policies emphasizing patient dignity, safety, communication, continuous training, teamwork, cooperation with other facilities, and sound management.

The evolution of BSC at Bethlehem Garden Hospital can be traced through five distinct periods.

### 3.1 Initial implementation (2016)

The hospital initiated BSC implementation following the appointment of Makoto Kikuchi as the new Administrative Director. While this marked an important strategic shift, the initial implementation faced challenges due to a lack of external guidance and clear strategy. This period highlighted the importance of proper preparation and strategic clarity before implementation.

### 3.2 Early operation (2017–2018)

The first BSC operation revealed several critical areas requiring improvement, including misalignment of the four perspectives, particularly in the causal link between patient and financial perspectives, insufficient objective alignment across departments, and lack of coherence among strategic themes, objectives, and indicators. Despite these challenges, the hospital demonstrated its commitment to transparency by publicly disclosing its BSC on its website. The BSC challenges identified at this period are detailed in Ito (27).

### 3.3 Enhancement period (2018–2020)

External expert engagement led to significant improvements through structured training programs, comprehensive strategy map development, and departmental BSC implementation. The hospital used a SWOT analysis to understand the current situation and then created a BSC and began rolling out departmental BSC across seven departments. These improvements corresponded with decreased turnover rates and increased employee engagement scores. Huang et al. (12) describe the training program in detail.

### 3.4 Integration period (2020–2022)

The BSC evolved to address broader organizational responsibilities by integrating SDGs and introducing new indicators for ICT implementation, environmental impact, and social responsibility. The hospital enhanced stakeholder communication through multiple channels (e.g., websites, blogs, PR magazines, conference activities, community interaction activities, questionnaire surveys), demonstrating how BSC can adapt to changing organizational needs while maintaining strategic focus. A discussion of the integration of the BSC and SDGs is detailed in Nemoto (28).

### 3.5 Maturation period (2022–2024)

The BSC reached a more sophisticated stage with explicit linkages to SDGs targets (link SDGs targets with BSC’s action plans), enhanced risk management indicators, and metrics for employee motivation. This period has resulted in a further decline in turnover rates, although future trends must be monitored.

This evolution demonstrates how BSC can transform from a basic performance measurement tool into an integrated tool for strategic management and communication. The hospital’s experience suggests that successful BSC implementation requires phased deployment, external expertise, continuous refinement, and strong stakeholder engagement, ultimately leading to significant organizational improvements.

One of the most significant outcomes observed following the implementation and refinement of the BSC was a substantial decrease in employee turnover rates. The data provided by the hospital shows a marked decline in turnover rates from 23.6% in 2015 (pre-BSC implementation) to 3.4% in 2023. This reduction is particularly noteworthy when compared to industry averages in Japan and internationally. Turnover rates are discussed in the next section.

The Administrative Director of Bethlehem Garden Hospital provided insights into the relationship between BSC implementation and the reduction in turnover rates. Kikuchi (40) noted that before BSC adoption, the hospital faced challenges with high turnover, particularly in the nursing department, which experienced rates in the 30% range. The recruitment landscape was difficult, often requiring reliance on recruitment agencies. New hires frequently struggled to acclimate to the organizational atmosphere, leading to swift departures and perpetuating a negative cycle of acceptance, disillusionment, and exhaustion.

The Administrative Director emphasized that the BSC served as an effective tool for visualizing and disseminating the organization’s values to all staff members. He stated, “The most valuable asset that can be attained from efforts to make values visible is ‘trust between the organization and staff.’ I believe this ‘relationship of trust’ has contributed considerably to the reduction in turnover.”

While a direct causal relationship between BSC implementation and reduced turnover rates cannot be definitively established, the case of Bethlehem Garden Hospital suggests a potential relationship. The BSC appears to have facilitated better communication of the hospital’s mission, vision, and strategy, promoted alignment between individual and organizational goals, and fostered a sense of trust and engagement among employees.

## 4 Discussion

In Japan, the MHLW has surveyed the turnover of the healthcare and welfare industry, and the JNA has surveyed nursing staff turnover. Data on hospital turnover in the United States will also be presented for reference (Table 1). These data present turnover rates from 2015 to 2023, the same period as the data from Bethlehem Garden Hospital. However, the JNA survey does not have data for 2015 and 2016. Thus, the 2014 data is included for reference. The JNA has not yet released data for 2023.

**Table 1:** Comparison of turnover rates.

Years	Bethlehem Garden Hospital	Healthcare and welfare industry	Full-time nursing staff	Hospital average in the United States
2015	23.6%	14.6%	None (2014: 10.8%)	17.1%
2016 (BSC implementation)	15.4%	14.9%	None	16.2%
2017	15.6%	14.5%	10.9%	18.2%
2018	13.1%	15.5%	10.7%	19.1%
2019	12.0%	14.4%	11.5%	17.8%
2020	8.7%	14.2%	10.6%	19.5%
2021	7.8%	13.5%	11.6%	25.9%
2022	9.0%	15.3%	11.8%	22.7%
2023	3.4%	14.6%	None	20.7%

Source: Prepared by authors based on materials provided by Bethlehem Garden Hospital; (40–44).

Table 1 shows that the healthcare and welfare industry turnover rate is around 14%, while the nursing staff turnover rate is around 11%. Therefore, Bethlehem Garden Hospital had a higher-than-average turnover rate until 2018 (If nursing staff turnover is to be used as a comparison, 2019). However, since then, it has had a lower-than-average turnover rate. Compared to the turnover rate of the United States hospitals, Bethlehem Garden Hospital’s turnover rate in 2015 was 23.6%, much higher than the United States turnover rate (17.1%). However, after 2016, when Bethlehem Garden Hospital implemented the BSC, the turnover rate was lower than that of the United States.

In summary, Bethlehem Garden Hospital’s turnover rate has changed from being higher than the average turnover rate in Japan and the United States to being lower since the introduction of BSC. As noted earlier, turnover in healthcare is an important indicator related to quality of care and patient safety. This could be a piece of evidence that the BSC has an impact on the quality of care and patient safety.

The Administrative Director cites employee motivation as a factor associated with lower turnover. In other words, he points to a relationship with employee engagement. Bethlehem Garden Hospital has been conducting an employee survey (excluding those in positions of chief or higher) since 2015. This survey asked questions related to employee engagement. Table 2 presents figures on turnover rates and employee engagement. These figures are based on the questions “Do you feel motivated and happy at work?” (2015–2017) and “Do you take pride in your work?” (2019–2023), based on the percentage of positive responses to these questions. Note that the 2018 numbers are “None” because the survey was not administered in 2018.

**Table 2:** Comparison of turnover rates and employee engagement scores in the Bethlehem Garden Hospital.

Years	Turnover rates	Employee engagement scores
2015	23.6%	72.1%
2016 (BSC implementation)	15.4%	74.2%
2017	15.6%	74.1%
2018 (Action research begins)	13.1%	None
2019 (Training programs and departmental rollout begins)	12.0%	84.1%
2020	8.7%	89.8%
2021	7.8%	89.9%
2022	9.0%	86.3%
2023	3.4%	87.9%

Source: Prepared by authors based on materials provided by Bethlehem Garden Hospital; Kikuchi (40).

As shown in Table 2, employee engagement scores improved at Bethlehem Garden Hospital, along with a decrease in turnover. This is similar to previous studies [e.g., (29–34)] showing that employee engagement leads to lower turnover. Some of these studies point to the impact of communication on employee engagement. In other words, they suggest that using the BSC as a communication tool can encourage employee communication, which can lead to increased engagement. Higher employee engagement scores may improve the quality of care (35).

The trend of high engagement scores needs to be interpreted as a ceiling effect. The ceiling effect refers to the phenomenon in which measurements are concentrated near the upper end of the scale (36). Gallup, Inc. (37) shows that even best-practice organizations typically maintain engagement scores around 70–73%, with their highest recorded score being 73% in 2020. In light of this, the hospital’s scores of 86–90% are remarkably high, and the subsequent stability at this level suggests that employee evaluations of the organization have crossed a certain threshold and have reached a stable state. This is particularly notable given that Japanese people generally tend to have low engagement scores (38). Although not an ongoing survey, Gu & Itoh (39) noted through a 2013 survey that 83% of Japanese physicians, 61% of pharmacists and technicians, and 31% of nurses were satisfied with their jobs. While exact comparisons are difficult to make due to differences in measurement methods and timing, these benchmarks suggest that the engagement scores of the Bethlehem Garden Hospital are exceptionally high in the Japanese healthcare context.

Also noteworthy is the change from 2019 to 2020: as described by Huang et al. (12), Bethlehem Garden Hospital introduced a training program with external BSC experts as part of its communication management starting in 2019. Since 2019, when the new communication program was introduced, there has been an accelerated improvement. The turnover rate decreased from 12.0% in 2019 to 8.7% in 2020 and further to 7.8% in 2021. Similarly, engagement scores also showed marked improvement: after hovering around 74% from 2015 to 2017, scores rose to 84.1% in 2019 and continued to improve, reaching 89.8% in 2020 and 89.9% in 2021. The latest data available for 2023 shows a turnover rate of 3.4% and an engagement score of 87.9%. This could be strong evidence supporting the usefulness of communication management using the BSC. However, the departmental rollout of the BSC under the guidance of an external expert has also taken place in conjunction with the introduction of the training program. Therefore, the possibility of an interaction effect on turnover and engagement should be considered.

These discussions indicate that the Bethlehem Garden Hospital case study demonstrates the importance of communication in healthcare institutions and the usefulness of the BSC as a communication tool.

## 5 Conclusion

This study examined the role of the BSC as a communication tool in addressing employee turnover at Bethlehem Garden Hospital in Japan. The findings suggest that the implementation and improvement of the BSC coincided with a significant decrease in turnover rates, from 23.6% in 2015 to 3.4% in 2023. This reduction is particularly noteworthy when compared to the average turnover rate in the Japanese healthcare and social services industry (approximately 14%) and the turnover rate of nursing staff (approximately 11%). The case study highlights several key points:

1. The BSC, when properly implemented and communicated, can serve as an effective tool for aligning organizational goals and fostering employee engagement.
2. The reduction in turnover at Bethlehem Garden Hospital corresponded with an increase in employee motivation and engagement scores, supporting previous research on the relationship between engagement and retention.
3. The use of the BSC as a communication tool can contribute to improved organizational trust and employee understanding of the institution’s mission, vision, values, and strategy.

By fostering improved communication, alignment of goals, and employee engagement, the BSC may offer a promising approach to improving workforce stability and, ultimately, the quality of care.

While these findings are promising, it is important to note the limitations of this study. As a single case study, the results may not be generalizable to all healthcare settings. In addition, while the potential relationships between BSC implementation and reduced turnover are strong, causality cannot be definitively established without further studies. It should also be noted that the study is based on the Japanese context. Different organizational contexts, cultures, and resource availability could affect BSC implementation and outcomes. Furthermore, data collection was partially constrained by COVID-19 restrictions, necessitating adaptation of research methods, particularly in the later stages of the study.

Future research directions may include comparative studies across multiple healthcare institutions to validate the impact of BSC on turnover rates; longitudinal studies to assess the long-term effects of BSC implementation on employee retention and engagement; qualitative research to explore the specific mechanisms by which the BSC influences employee perceptions and behaviors; exploration of potential cultural or organizational factors that may affect the effectiveness of the BSC in different healthcare settings.

Despite these limitations, this study makes several important contributions. Theoretically, it extends our understanding of how strategic management tools can enhance communication in the organization. Practically, it provides insights into the effective implementation of BSC in healthcare settings, particularly for addressing critical issues such as employee turnover and engagement. The study also demonstrates the potential of integrating global sustainability goals into local organizational practices through BSC implementation.

## Data Availability

The original contributions presented in the study are included in the article/supplementary material, further inquiries can be directed to the corresponding author.
